# Entrepreneurship and innovation in Nigerian universities: Trends, challenges and opportunities

**DOI:** 10.1016/j.heliyon.2024.e29940

**Published:** 2024-04-23

**Authors:** Muyiwa Oyinlola, Oluwaseun Kolade, Silifat Abimbola Okoya, Olubunmi Ajala, Arinola Adefila, Adedapo Adediji, Kunle Babaremu, Bosun Tijani, Jude Adejuwon, Faith Wambui, Esther Titilayo Akinlabi

**Affiliations:** a Institute of Energy and Sustainable Development, De Montfort University, Leicester, LE 1 9BH, UK; b Sheffield Business School, Sheffield Hallam University, Sheffield, UK; c School of Economics, Finance and Accounting, Coventry University, CV1 5DL, UK; d Staffordshire Centre for Learning and Pedagogic Practice, ST4 2DE, UK; e Directorate of Pan African University Life and Earth Sciences Institute (PAULESI), Ibadan, Oyo State, Nigeria; f Co Creation Hub, 294 Herbert Macaulay Way, Yaba, Lagos, Nigeria; g iHub, Galana/Lenana Road, Nairobi, Kenya; h Department of Mechanical and Construction Engineering, University of Northumbria, Newcastle Upon Tyne, UK

**Keywords:** Entrepreneurship education, Innovation, Nigeria, Entrepreneurial aspirations, Entrepreneurial outcomes, University-industry partnership

## Abstract

In a bid to improve entrepreneurial outcomes of graduates from Nigerian universities, the Nigerian government has launched a range of interventions, including a 2004 national policy mandating compulsory inclusion of entrepreneurship education in the curriculum, and support for the establishment and implementation of entrepreneurship development activities by university departments. However, not much is known about the operational nuances, challenges and impact cases that characterise the implementation of this national policy in such a vast, culturally diverse country as Nigeria. To address this lacuna, this study draws on qualitative data from five focus groups, crystalised with quantitative data from 151 students across four Nigerian Universities, to explicate the current trends, successes, and challenges of entrepreneurship development and innovation support in Nigerian universities. The paper offers recommendations on how the current policy on entrepreneurship education in Nigeria can be enhanced to improve student entrepreneurial aspirations and outcomes. It also signposts innovative pedagogic activities which could be deployed to achieve this.

## 1 Introduction

Historically, entrepreneurs have played a variety of roles in the economic development of nations. They are often described as disruptive innovators who are quick to recognise opportunities and take risks to access and harness them in a process of creative destruction [1]. According to Kennard [2], “entrepreneurs are innovators and innovators can be entrepreneurs” [2, p. 12]. The entrepreneur is in constant search for, and exploitation of, innovative ideas and viable business opportunities [3,4], some of which have the potential to transform societies. Most entrepreneurs have a natural instinct which is either cultivated organically in the business climate or developed through the formal educational system [5]. To foster future entrepreneurs, it is important to ‘catch them young’. Many scholars have argued that participating in entrepreneurial education impacts students' resolve to be entrepreneurial and become a success [6,7]. Some universities have therefore evolved to adopt a modern purpose to positively empower graduates to be changemakers, leaders and entrepreneurs equipped with the competencies, attributes, and attitudes to influence economic and social development [8].

For African economies such as Nigeria, where the youth unemployment rate is purported to be an average of 42.5 % [9], integrating entrepreneurship education into the curriculum of universities prepares graduates to effectively contribute to society [10]. Entrepreneurship education has been recognized as a tool for curbing unemployment and supporting sustainable development [11,12]. It has been introduced widely and started taking prominence in Nigeria in 2006 [13]. Adejimola and Olufunmilayo [14] reported that approximately 80 % of graduates from Nigerian universities struggle with obtaining employment partially due to the curricula that focuses on obtaining ′white-collar jobs’. In response to this, the Federal Government through the National Universities Commission (NUC) and Industrial Training Fund (ITF) have accelerated efforts to drive entrepreneurship education in Nigeria. This involves a directive for higher institutions of learning in Nigeria to include entrepreneurship education as a compulsory course into the curriculum [15,16]. This in turn has led to most institutions establishing entrepreneurship development centres to support embedding of targeted interventions [10] and empowering graduates with appropriate skills. The Federal Government of Nigeria also established some schemes in partnership with stakeholders to enhance entrepreneurship skills amongst Nigerian students and graduates. The agencies tasked with supporting the schemes are the National Directorate of Employment (NDE), Student Industrial Working Experience Scheme (SIWES), Vocational and Technical Training, Agricultural training, and Information and Communication Technology (ICT) Training [[15], [16], [17]]. These initiatives are designed to be inclusive, ensuring learners from all backgrounds access the life skills and opportunities which support entrepreneurship [10,18,19].

The evaluation of the interventions has been weak [16,18] and anecdotal evidence garnered during project conversations from the perspective of students and academics indicates several gaps in enacting the policy at local level. Therefore, this study aims to fill this gap by providing insights into the current trends, successes and challenges of entrepreneurship and innovation in Nigerian universities. It makes an important contribution by illuminating factors that affect entrepreneurship aspirations in students. This study is significant because it provides information on how the current policy on entrepreneurship education in Nigeria can be enhanced to improve student outcomes as well as identifying which pedagogic activities could be deployed to achieve this.

The rest of the paper is structured as follows. Section 2 presents a literature review on the subject highlighting the link between entrepreneurial education and national economic development, using a triple helix model to unpack the dynamic interactions among university, industry, and government actors. This is followed by a description of the empirical context of the study. Section 3 presents an in-depth description of the methodology and sample employed in this study. Section 4 presents the results from the quantitative and qualitative study. Section 5 discusses the results with respect to policy and pedagogic implications and section 6 outlines the main conclusions, limitations of the study and suggests areas for further research.

This manuscript represents a segment of a larger inquiry into innovation and sustainable development practices among Nigerian students, and it does not encompass the full range of our extensive dataset. To provide a clear and focused analysis, we have compartmentalised our findings. Thus, this paper delves into the role of entrepreneurship in Nigerian universities—a reflection of current national educational policies and their impact on job creation. Meanwhile, a separate paper [20] explores the students' engagement with the circular plastic economy, a specific sector and emerging area not yet prioritized by government policy. By partitioning our analysis, we avoid diluting our narrative and ensure that each research strand receives dedicated attention. This bifurcated presentation enables a comprehensive and lucid exploration of distinct but interrelated themes, in line with the varying hypotheses and objectives that underlie our broader research project.

## 2 Literature review

### 2.1 Background

In 1732, Richard Cantillon described ‘entrepreneurship’ as "a willingness to carry out forms of arbitrage involving the financial risk of a new venture" [20, p. 603]. Since then, various definitions of entrepreneurship have been postulated. In any case, entrepreneurship can be described as the process of making, initiating, building, and growing sustainable businesses while grooming a team that can harness available resources as well as exploring marketplace opportunities for long-term wealth and financial gains [21].

On the other hand, the word ‘innovation’ has its origins from the Latin “innovare” which means renewal [22]. Various definitions of innovation are proposed in the discourse [23] and a multifaceted nature of using the terminology is dependent on local norms, ontologies, and practices [24]. Innovation serves as an important driver for economic growth and development in various facets of the economy such as education, commerce, transportation, and telecommunication [25]. To ensure viability, businesses around the world are adopting innovative strategies that focus on investing in trainings, research and development, firm size, type, and sector [26].

### 2.2 Entrepreneurship and innovation in universities: a triple helix perspective

Entrepreneurship and innovation have progressively become popular in the world of academia, with universities seen as key players in driving economic growth through the creation of new businesses and the development of innovative technologies [27]. Entrepreneurship education has been reported to support an increase in the value of entrepreneurial outputs [28,29], entrepreneurial skills are also promoted to ensure that university students develop an opportunity-oriented perspective [30]. In recent times, several national governments have increased collaboration with universities in a bid to drive initiatives for entrepreneurship and innovation [31]. In Singapore, for example, the economy has been in a consistent state of reformation since their independence, with particular attention on the manufacturing sector. The reforms focused mainly on a model that was geared towards the transformation of the universities to ensure promotion of entrepreneurship and innovation in academia via partnership with industry, in the triple helix model of innovation [32]. This synergistic approach consolidated university-industry-government collaboration in business innovation and technological advancement and facilitated the attainment of the national agenda of entrepreneurship and innovation [33,34].

The Triple Helix has become a popular institutional arrangement model that has proven most suited to the development of entrepreneurship and economic growth compared with competing ideas and theories. The Triple Helix's basic tenet is that the knowledge sector's growing influence has sparked new kinds of interactions between the institutional domains of government, industry, and academia, which were previously distinct and frequently isolated [34,35]. These dynamic interactions have given rise to an institutional framework that is integrative and crosses boundaries in which “industry operates in the Triple Helix as the locus of production; government as the source of contractual relations that guarantee stable interactions and exchange; and the university as a source of new knowledge and technology, the generative principle of knowledge-based economies” [36, p. 295]. The Triple Helix is thus characterised by the emergence of hybrid organisations at overlapping institutional spheres [36,37].

The possibility of addressing two complimentary issues - the need for students to gain practical experience and the need for entrepreneurs to access fresh knowledge that might boost their competitiveness and productivity - is provided by university-industry partnerships [38]. According to the logic of hybrid, boundary spanning collaborations, neither industry players nor academic institutions are the sole creators or consumers of information. Industry professionals give students the chance to gain real-world experience that cannot be replicated in a classroom. Furthermore, university-industry partnerships present aspiring and budding entrepreneurs a place to test and hone their ideas/concepts in the day-to-day crucible of entrepreneurial graft [39]. Universities play a key role in the creation and execution of entrepreneurship education programmes due to their historical placement as the principal hub of knowledge production [40]. The collaborative process that gathers and combines important inputs from industry stakeholders and policymakers is facilitated and coordinated by universities [41,42].

Russia had various systems of production which did not have many mechanisms for innovation; in the last decade, there has been policy interventions to rekindle innovation and encourage an entrepreneurial culture with universities playing an active role in initiating entrepreneurship education [24]. Government has provided support for this scheme, although there is still a lot to be done, the results have been inspirational with the development of innovation infrastructure and moves towards an entrepreneurial culture [24]. Similarly, universities in Brazil have done a substantial amount of work developing a ‘social mission’ which aligns with the notion of social innovation and entrepreneurship. However, there has been some criticism on the dualism of universities trying to be ‘entrepreneurial’ while also assuming roles of social responsibility by being ‘engaged’, especially in developing regions amidst challenges of extreme inequality and poverty [43]. In India, entrepreneurship development has been a response to problems of unemployment and poverty within a context of safeguarding inclusive growth, especially in training institutions. However, it has been said that there is a need to assess the impact of economic change through the promotion of entrepreneurship, strategies in relation to creation of adequate environments for teaching and training of entrepreneurship as well as the role of institutions in developing entrepreneurs [44].

Several universities on the African continent have engaged in facilitating entrepreneurship and innovation [45]. South Africa has a University of Technology with approximately 17,000 students and 300 programmes with an academic emphasis on career-focused diplomas to achieve its vision of leading innovative knowledge and technical education [46]. In post conflict settings such as Rwanda, entrepreneurship in academia has a critical role to play in nation building of civic society and organisations to prevent a reoccurrence [47]. However, there have been limited studies on the impact while emphasis has been placed on the need to balance the achievement of socioeconomic needs and foster integration [48].

### 2.3 Entrepreneurship and innovation in nigerian universities

The Federal Ministry of Education, which has various parastatals for regulating and accrediting all types of educational institutions, oversees the Nigerian education sector. All universities in Nigeria are subject to regulation and accreditation by the National Universities Commission (NUC) [49]. The fundamental objectives of Nigerian universities, as stated in the National Policy on Education released in 2004 [50], include developing workforce, intellectual values and talents, physical and knowledge skills, scholarship and community service, national unity, and intercultural contacts.

Nigeria, as one of the leading economies in Africa, has recognized the importance of entrepreneurship and innovation in driving economic development, and Nigerian universities are increasingly being tasked with promoting these activities. According to Ref. [17], several factors have hindered entrepreneurship education in Nigeria. However, there have been some positive developments in recent years. For example, the Nigerian government has launched several initiatives aimed at promoting entrepreneurship and innovation in universities, such as the Presidential Entrepreneurship Summit and the National Science, Technology, and Innovation Roadmap (NSTIR) [51]. These initiatives are designed to encourage universities to collaborate with industry and to provide funding for research and development activities.

The concept of entrepreneurship education and its application for graduates of Nigerian universities cannot be over-emphasized and several scholars such as [11,17,52] explored this concept. Drawing on quantitative data from 6 Nigerian Universities [53], highlighted the relation between three types of Entrepreneurial Education (formal, informal, and non-formal) on entrepreneurship intentions of students and observed that only the formal entrepreneurial education had an impact on student intentions. Okolie et al. [54] conducted a study with 1191 participants from 12 Nigerian universities to assess the entrepreneurial competencies of final year undergraduate students. They observed that students’ participation in the current compulsory entrepreneurship education positively influenced five of the thirteen entrepreneurial competencies. They further recommended entrepreneurship programs should be practical based rather than theory based. Otache et al. [55] conducted a quantitative study using 217 participants from 3 Nigerian Universities and observed the entrepreneurial orientation, entrepreneurial motivation and entrepreneurial intentions of students increased significantly after being exposed to Entrepreneurship Education. Otache et al. [56] employed the theory of planned behaviour to assess the effects of entrepreneurship education on 250 Nigerian students and found exposure to entrepreneurship education resulted in improved outcomes.

Our literature review has underscored the fact that entrepreneurship and innovation within Nigerian universities has become a crucial element of the academic discourse. Our study therefore aims to contribute to the literature by providing insights, into the current trends, successes and challenges of entrepreneurship and innovation in Nigerian universities. Furthermore, this paper provides empirical insights that can inform policy formulation, curriculum development, and institutional strategies to bolster this sector, which is paramount for national development.

## 3 Material and methods

The paper employed a mixed-methods approach [57] to comprehensively collect and analyse data, combining quantitative insights from a survey of university students with qualitative findings from focus group discussions. The quantitative data enable us to examine the relationship among the key variables of interest. However, as the survey questions are close ended, they are inadequate to capture the complexity of respondents' feelings and thoughts and the situational nuances that underpinned their experiences. Furthermore, cross-sectional surveys provide a snapshot of a specific moment in time and do not account for changes over time, a factor that is important in any behavioural study. Thus, to mitigate these weaknesses, we followed up the cross-sectional survey with focus group discussions to deepen the understanding of students' experiences of entrepreneurship education programmes, and how the design of pedagogical activities or initiatives shape their motivation and decision to engage in entrepreneurial action. This complimentary integration of quantitative and qualitative approaches enhances the crystallisation of data to refine and interpret themes, thereby enriching the understanding of the participants' experiences and perspectives [58]. Crystallisation encourages the researcher to gather information from a variety of sources and consider these from diverse perspectives to construct a representation of the participant's reality. Crystallisation goes beyond the triangulation approach that focuses on simplistic, two-dimensional components [59]. Details of the methods are presented below.

### 3.1 Quantitative methods

#### 3.1.1 Data collection

A student survey, that was administered electronically, was used to gather quantitative data. The respondents were recruited through a purposeful sampling approach as all respondents were participants of the British Council Circular Plastic Economy Innovation (BC-CPEI) Hub initiative. The initiative was designed to deliver a series of training workshops to students from 4 universities; University of Lagos (UNILAG), in the commercial capital of the country, which has 57,000 students, the Obafemi Awolowo University (OAU) in the West, which has 30,000 students, the Amadu Bello University (ABU) in the North with 49,954 students, and the University of Nigeria, Nsukka (UNN) in the East, which has 36,000 students. The four universities chosen are a good geographical and regional representation of Nigeria. Also, they represent four of the five institutions known as first-generation universities (the fifth being the University of Ibadan) [60]; as Nigerians fought for local education throughout the colonial era freedom battles, three of these universities were born out of the Education Ordinance of 1952 [61]. Nearly every other university in Nigeria is connected to these elite institutions, either directly or indirectly.

Professors, university departments, social media profiles, and various university WhatsApp groups were used to invite students to participate in the BC-CPEI hub initiative. In order to ensure that the cohort was balanced across gender, study level, and course of study, applicants were asked for their basic demographics, contact information, and field of study or specialism. 151 candidates were selected based solely on their commitment to adhere to the training regimens. It should be noted that the training programme coincided with an eight-month closure of most Nigerian universities due to industrial action by academic staff, which notably constrained the recruitment process. Nevertheless, all participants who expressed a willingness to engage were included in the programme.

Ideation workshops were hosted between March and April 2022, in which participants were given the URL of a Google Form at the beginning of the workshop. The questionnaire was designed to capture students' perception on their individual entrepreneurial competencies and aspirations using a 5-point Likert scale [62]. The questions were framed based on key concepts and principles from the literature relating to innovation and entrepreneurship education in university environments [12,63,64]. All 151 students responded to the survey which is about 0.1 % of the total population (172,954) of students enrolled in all four universities combined.

The main dependent variable, entrepreneurial intention, was operationalised using two items in the questionnaire, on a Likert scale of 1–5, from strongly disagree to strongly agree: *After my studies, I would like to start my own business;* and then: *I want to start a business that contributes to the circular plastic economy*. These capture intentions relating to entrepreneurship as well as focusing specifically on circular economy entrepreneurship.

The predictors in the model are listed as follows, all of them measured as Likert scale items on a scale of 1–5, from strongly disagree to strongly agree (see supplementary material):

PBExp: Previous business experience.

Aweb: awareness of web applications.

BPlan: business planning skills.

For controls, we incorporated gender, age and university attended into the model.

Table 1 shows the profile of the respondents and the questionnaire used is presented in the supplementary material.

**Table 1:** Profile of Questionnaire respondents.

Variable		Frequency	Percent
Gender	Male	103	68.2
	Female	48	31.8
	**Total**	**151**	**100**
Age	18–24	71	47
	25–34	56	37.1
	35–44	17	11.3
	45–54	7	4.6
	**Total**	**151**	**100**
University	Ahmadu Bello University	56	37.1
	University of Nigeria	23	15.2
	University of Lagos	40	26.5
	Obafemi Awolowo University	29	19.2
	Other^a^	3	2.0
	**Total**	**151**	**100**
Current Level of Study	Post PhD	5	3.3
	PhD	17	11.3
	Masters	44	29.1
	Undergraduate	82	54.3
	Others^a^	3	2.0
	**Total**	**151**	**100**

a The other category was used to capture the 3 students who listed universities other than the four universities that were used in this study. This could be students who were shortly progressing to other universities, hence indicated their soon to be universities.

#### 3.1.2 Data analysis

The responses from the questionnaire were input into SPSS (Statistical Package for the Social Science) and descriptive statistics were used for preliminary exploration of the data. Major data cleaning was unnecessary, as the questionnaire design included pre-emptive data validation that required all fields to be completed before submission, ensuring that respondents provided a full set of responses. However, for the purposes of the final analysis in this paper, we omitted certain questionnaire items that, while pertinent to the broader BC-CPEI project, were not directly relevant to the specific research aims of this paper. Further analysis was done to provide insights on the demographics of the participants and explore any relationship between trends observed and socio-cultural factors such as gender and location. An extended multinominal logistic regression analysis was conducted to assess factors that may affect the odd ratios of individuals in Nigerian universities being, interested in starting a business after completing their studies. This dependent variable was measured using the Likert scale responses (“Strongly Agree,” “Agree” and “Disagree”) to the question: “After my studies, I would like to start my own business.” The original question has five categories, we however recoded into three (merging “Disagreed” and “Strongly Disagreed”) because only 2 respondents strongly disagreed. This is to avoid miscalculating the standard errors for the “Strongly Disagreed” category.

Algebraically, our estimation outcome corresponds to:

$$Is\left(\frac{P\left(StarBiz=\text{Agree}\right)}{P\left(StarBiz=\text{Strongly}\;\text{Agree}\right)}\right)=X_{i}^{j}\beta_{k}+\varepsilon_{i}$$

$$Is\left(\frac{P\left(StarBiz=\text{Agree}\right)}{P\left(StarBiz=\text{Strongly}\;\text{Agree}\right)}\right)=X_{i}^{j}\beta_{k}+\varepsilon_{i}$$

Where: $X^{J}\text{:}$ is K X 1 vector of explanatory variables

Β: is the conformable vector of coefficients.

The baseline model (Model 1) controls, for the effect of age, gender, and the university (which may be interpreted also, as their location). The second model (Model 2) controls for the initial business experience of individuals in our observations. The third model is extended to assess the likely effect of their awareness of innovations (Web Application) may have on the odd ratios of being interested in starting a business after their studies. The final model (Model 4) controls, for entrepreneurial skills (their self-assessment of their ability to prepare a business plan).

Explicitly:

Model 1 (baseline):

$X^{J}\text{:}$ consists of Age, Gender, and University.

Model 2 $X^{J}\text{:}$ consists of Age, Gender, University and Previous business experience

Model 3 $X^{J}\text{:}$ consists of Age, Gender, University, Previous business experience and Awareness of innovation (Web App).

Model 4 $X^{J}\text{:}$ Consists of Age, Gender, University, Previous business experience, Awareness of innovation (Web App) and Entrepreneurial skills (Business Plan) and epsilon represents the random error which we assume to be well-behaved in this study.

### 3.2 Qualitative methods

#### 3.2.1 Data collection

Qualitative data was collected through focus group discussions [65]. Student participants for the focus groups were identified from those who actively participated in the ideation workshop. Selection was based on their engagement level and demonstrated ability to contribute valuable insights to the discussion. For the selection of lecturers, a snowball sampling approach was employed across the four universities. Academics involved in the BC-CPEI hub initiative extended invitations to their colleagues, thus leveraging professional networks to enrich the diversity of the focus group discussions. A total of five focus group discussions (FGD) were held using Microsoft Teams and Audio recorded. Findings from the literature review guided the discussion prompts, shown in the supplementary material, that were prepared in advance. Details of the focus groups is presented in Table 2.

**Table 2:** Details of focus groups.

Focus group	Number of Participants	Description
Obafemi Awolowo University - Staff and Students	8	Staff and students from various departments of Obafemi Awolowo University.
University of Lagos – Staff	6	Staff from various departments of University of Lagos
University of Lagos - Students	5	Student entrepreneurs from University of Lagos
University of Nigeria - Staff	4	Staff from University of Nigeria
Finalists of Innovation challenge	6	Students from Ahmadu Bello University, Obafemi Awolowo University, University of Nigeria, University of Lagos. Participants were finalists of the innovation challenge hosted by the circular plastic innovation hub

#### 3.2.2 Analysis

The audio recording of the responses from the focus groups were transcribed and then analysed using NVIVO 13 software. Braun and Clarke's [66] six stage reflexive thematic analysis were used for the analysis process - (i) familiarisation with the data, (ii) coding the data (iii) generating initial themes using Behavioural Mapping tools (iv) reviewing and developing themes (v) refining, defining and naming themes (iv) producing the report.

Behaviour mapping tools are used for analysis to observe individual or group behaviour within a specifically defined research environment [67]. There are a number of challenges and strengths for using behaviour analysis in education evaluation research. The tool enhances the research process by providing an opportunity to evaluate participants behaviour and analyse how this has may have an influence on the phenomena in question. The research project focused on interrogating how entrepreneurial education activities and investments in learning environments with the Nigerian Higher Education sector had an influence learners' skills and entrepreneurial behaviour. The behaviour mapping also provided an instrument for examining spatial features and interactive dimensions which supported or acted as a barrier for developing entrepreneurial education. A deductive approach was used to map how institutions were supporting or investing in entrepreneurial education. Using principles from educational effectiveness research [68], the project explored how the learning environments are adapting pedagogically and structurally to the use of interactive resources, digital learning objects and collaboration with stakeholders in business, industry, government and third sector.

The Pedagogy-Space-Technology-User framework [68,69] was used as themes were generated to map the behaviours of the participants [70].Firstly, the transcripts were organised and reviewed in search of important textual information in respondents’ responses and opinions. The process enhanced familiarity with words, phrases or clauses which connoted the same meaning. Secondly, iterative manual coding with multiple stage categorization of data points was employed. The first set of pattern codes was made using words, phrases, and clauses. Behaviour mapping was used to support the generation of initial themes around educational entrepreneurship in the Nigerian HE context. The tool provided insights around how participants engage in the behaviours of entrepreneurship education. The pattern codes were then reviewed to make phrases, clauses, and sentence patterns for thematic naming and identification. The themes were then refined, defined, and categorised into the names used for interpreting the results. This approach has been used widely for qualitative data analysis [70].

### 3.3 Ethics

The present study was approved by the Faculty of Health & Life Sciences Faculty Research Ethics Committee at De Montfort University with approval number 3927. Consent for participating in the survey was obtained electronically as part of the questionnaire, and verbal consent was audio recorded during the focus groups. The decision to collect verbal consent for the focus group discussions was primarily influenced by the logistical challenges of conducting these sessions online. Given that our focus groups were held virtually, obtaining written consent would have required participants to download, sign, scan, and upload documents. This process was deemed impractical, especially considering the pay-*as*-you-go nature of internet access in Nigeria, where users are particularly conscious of their data consumption. To ensure ethical compliance and document consent effectively, we opted for verbal consent, which was audio-recorded at the start of each focus group session. This method allowed us to capture participants' consent efficiently while respecting their data limitations.

## 4 Results

Qualitative data is useful to describe, explain, and interpret human experiences [71].The data we have analysed enhances the insights provided by the quantitative data enabling us to understand how participants are experiencing the Higher Education ecosystem in Nigeria in relation to policies aimed at developing entrepreneurship and innovation. The qualitative comments have been interpreted using a behaviour mapping framework which provides an indication of the participants' behaviours and justification for what they value. The focus group discussions revealed some initial insights which were coded and segmented into three main categories – Opportunities, Challenges and Aspirations, as shown in Table 3. The key themes are examined to demonstrate the interventions that are effective in the contexts of participants, who also discuss how to better inform policy and practice.

**Table 3:** Insights from focus groups.

Category	Code	Description
**Opportunities for Entrepreneurship and Innovation Skills Development in Nigerian Universities**	OEID 1	Promotion of an enabling academic environment to foster research and collaboration to support potential entrepreneurs.
	OEID 2	Encouragement by some academic departments to promote teamwork and pitching of business ideas.
	OEID 3	Backing of supervisors, lecturers, and mentors to apply for entrepreneurship and innovation opportunities.
	OEID 4	Competitions linked to prizes urges students to think out-side-the-box.
	OEID 5	Incentives should be given to organisations that foster the development of innovative ideas individually or as teams.
	OEID 6	Students have developed ideas due to their collaboration and association with some organisations.
	OEID 7	University communities nurture good people networks for entrepreneurs that could facilitate idea generation.
	OEID 8	WhatsApp is an effective communication channel for amplifying information and ideas on campus using various closed groups.
	OEID 9	Business ideas on SDGs (Sustainable Development Goals) attainment and the sustainability of the environment should be encouraged to grow the community.
	OEID 10	Events in schools where professional on LinkedIn and entrepreneurs are invited to talk to students about entrepreneurship to inspired them to be entrepreneurs.
**Challenges for Entrepreneurial and Innovation Skills Development in Nigerian Universities**	CEID 1	Few departments teach entrepreneurship therefore lecturers' skills need to be developed to teach students.
	CEID 2	Some existing entrepreneurship courses are ineffective and feel like tick box exercises.
	CEID 3	Learning is mostly limited to classrooms with limited opportunities for peer-to-peer learning as well as mentorship by industry actors.
	CEID 4	Lack of information on opportunities to engage and develop.
	CEID 5	Lack of platforms to facilitate the development of innovative ideas.
	CEID 6	Dearth of the right entrepreneurship mindset, therefore translating viable ideas to reality becomes a challenge due to lack of practical skills.
	CEID 7	Lack of adequate contact and University synergy with external stakeholders such as industry players and entrepreneurs.
	CEID 8	Insufficient exposure to external conferences, competitions, etc.
	CEID 9	Lack of funds to scale innovation and seed enterprises
**Entrepreneurial Aspirations of Students**	EAS 1	Persistent academic strikes in Nigeria have driven entrepreneurial thinking amongst students to survive.
	EAS 2	Early entrepreneurship exposure from family and environments have developed business mindsets and aspirations in some students.

For the quantitative data, we adopted a hierarchical regression model where the control variables, gender, age, and university location were incorporated as regressors in the first model. In the second, third and fourth model, we progressively added each of three independent variables: previous business experience, awareness of web applications, and business planning skills. This approach enables us to evaluate more clearly the individual contributions of these predictors to the outcome variable of interest.

### 4.1 Opportunities for entrepreneurship and innovation skill development in nigerian universities

The results showed that Nigerian universities have responded positively to the mandate by the NUC (National Universities Commission) in terms of embedding Entrepreneurship Education across all undergraduate disciplines. All staff members that participated in this study were aware of the policy and showed a positive disposition to the policy as they agreed there was a need to foster entrepreneurship and innovation in higher education institutions.

Participants from all universities in this study highlighted that the NUC mandate was being fulfilled by university wide modules specifically designed to teach entrepreneurship to students. However, from a student perspective, these modules were not well valued, prioritized, or managed and most student respondents noted that they did not find them beneficial. They further highlighted that these were not well structured and properly thought out, leading to poor engagement by students. For example,

“It was only when **R2** mentioned the course on entrepreneurship that I remember that oh yeah, I had to write one exam at a time, on these.” (**FGD1-R4-Student**)

It was observed that some universities had gone beyond the policy to initiate other activities to foster entrepreneurship in students such as entrepreneurship development centres and hosting activities such as hackathons. The results indicated that asides from entrepreneurial education, there were other areas that were contributing to the development of entrepreneurial and innovation skills of students in Nigerian universities. Firstly, several participants who have started on the entrepreneurial journey highlighted that they acquired entrepreneurial and innovation skills through engagement in some extra curricula activities such as attending related programs, participating in related challenges and competitions, and participating in student association-related programs. However, few of the participants believed they acquired the necessary skills through personalized learning. Some of them mentioned the programs, association, and personal learning by saying:

“Okay, in addition to what R1 and R3 have said, I also want to give some credits to certain organizations at the university that has kind of helped, in a way.” (**FGD2-R4-Student**)

“… So, it’s like a combination of a formal and informal structure that comes up and at the end of the day.” (**FGD5-R3-Lecturer**)

Another intervention promoting entrepreneurial and innovation skills is the involvement of industrial partners in their studies, which gives them a hands-on experience and exposure to real life scenarios.

“… you see I quite of agree with her point of view that the engagement of entrepreneurs from the town (industry) is important.” (**FGD5-R6-Lecturer**)

A point that came out strongly was that through final year projects, a lot of students had developed innovations for local challenges:

“ I can quickly say that there are two of my past final year project student that are at the stage of getting NAFDAC (National Agency for Food and Drug Administration and Control) approval for their products that we did and there is also another student of mine who is trying to get approval from Standard Organisation of Nigeria, to come up with a product which was an idea that we designed during our final year project.” (**FGD5-R5-Lecturer**)

The participants further highlighted a hands-on research project work and business competition as a practice that fosters the acquisition of necessary skills needed for innovation and entrepreneurship. One respondent noted:

“For example, for two years now, almost every project I give my students must be tailored towards entrepreneurship …” (**FGD5-R4-Lecturer**).

Another respondent noted:

“So also, we like to encourage our students … For example, if we need to produce a composite material. So, what we do is that we try not to get the students to look into materials that are available within the country or within our local environment for them to research it. So that's how we try to go about it." (**FGD4-R2-Lecturer**)

The data suggests that the lecturers and curriculum have succeeded in raising awareness about entrepreneurial skills. The behaviours, experiences and commitments displayed show that learners are indeed developing the skills that are needed to support entrepreneurship. However there seems to be barriers with respect to translation of the practices, beyond graduation.

### 4.2 Challenges for entrepreneurial and innovation skill development in Nigeria universities

The results highlighted a few challenges facing entrepreneurial and innovation skill acquisition. Firstly, participants emphasized how difficult it was to source funds for scaling their innovations. Some of them reported that:

“Ok, so basically, to be frank, funding is needed. We are starting something and it is a challenge, frankly speaking.” (**FGD1-R6-Student**)

Secondly both staff and students highlighted the fact that while innovation was not a problem, the main issue was having the skills to take it to the next level. It was highlighted that these skills could not be taught but must be experienced.

Thirdly, lack of proper mentorship was identified as a challenge:

“So, those key roles [of mentorship] must be there in the mentor you are looking at: the information role, the power role and the development role.” (**FGD5-R5-Lecturer**)

Finally, the lack of support to progress students’ innovation to an enterprise, is another challenge that was highlighted. As one respondent noted:

“… most of them may have good ideas, most of them have developed something in their final year projects that they maybe want to use to make a living, but … they just end it there and join their mates in looking for jobs …” (**FGD3-R1-Lecturer**)

Similarly, another respondent noted:

"At least I've been working in this University for nine years now, and I haven't ever been asked to come for a meeting that this industry or this company has come to talk with our students. So, I'm talking about this episode from the standpoint of experience." (**FGD4-R4-Lecturer**)

### 4.3 Entrepreneurial aspirations of students

**Fig. 1:**
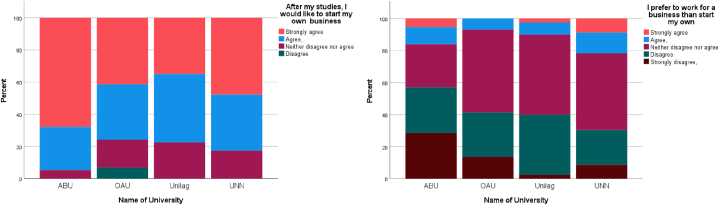
(a) Aspiration to start a business after studies (b) Comparing aspiration to work for a business to starting a business.

The results for the quantitative study were used to investigate the entrepreneurial aspiration of students. The quantitative results indicated that majority of students aspire to engage in entrepreneurial activities after graduation. Fig. 1a shows that in all 4 universities, more than 80 % of students would like to start their business after graduation. Similarly, Fig. 1b compares the desire of student to work for a business to starting a business. It can be observed that less than 20 % of students agree that they prefer to work for a business compared to starting a business, although a sizeable percentage (between 15 and 35 %) neither agree nor disagree.

The results showed that students were confident in their ability to develop innovative solutions:

“You know innovating for local challenges to solve a problem and for example R3 has told us innovation about how that peer-to-peer learning, he’s been able to develop innovation for peer-to-peer learning.” (**FGD2-R1-Student**)

Table 4 presents the result of the four models which aims to explain the entrepreneurial aspirations of students. For each of the models, we show the coefficients and standard errors of our explanatory variables for “Agree” and “Disagree” to starting a business after their studies relative to those who “Strongly Agree.” Our focus is in identifying those factors that may significantly affect the odd ratios of opting to start a business rather than predicting the marginal effects of each variable.

**Table 4:** Models’ results.

Variables	Model 1			Model4				
	Agree	Disagree	Agree	Disagree	Agree	Disagree	Agree	Disagree
Gender	−0.268	−0.438	−0.255	−0.431	−0.214	−0.475	−0.233	−0.476
	(0.425)	(0.527)	(0.426)	(0.529)	(0.431)	(0.531)	(0.436)	(0.533)
Age	0.230	−0.133	0.245	−0.102	0.250	−0.0975	0.328	−0.0518
	(0.246)	(0.369)	(0.248)	(0.374)	(0.249)	(0.374)	(0.256)	(0.378)
Univer	0.144**	0.213**	0.149**	0.227**	0.154**	0.225**	0.147**	0.222**
	(0.0678)	(0.0995)	(0.0684)	(0.102)	(0.0687)	(0.102)	(0.0697)	(0.103)
PBExp			−0.449	−0.985	−0.455	−1.011	−0.249	−0.875
			(0.454)	(0.695)	(0.455)	(0.697)	(0.473)	(0.709)
Aweb					−0.742	0.416	−0.888	0.309
					(0.669)	(1.175)	(0.692)	(1.182)
BPlan							0.397**	0.235
							(0.186)	(0.242)
Constant	−1.032	−1.360	−0.555	−0.328	0.770	−1.031	−0.516	−1.768
	(0.967)	(1.270)	(1.074)	(1.470)	(1.617)	(2.606)	(1.760)	(2.743)
Observations	148	148	148	148	148	148	148	148

Standard errors in parentheses.

***p < 0.01, **p < 0.05, *p < 0.1.

In all the four models, we find our variable “University” (which may also be interpreted for the location) as a consistent factor that affects the odd ratios of starting a business for the category that agreed to start a business and for the category that disagreed to starting a business, relative to those who strongly agreed. We find it to be statistically significant at 5 %. Our final model, however, also shows that our proxy for entrepreneurial skills (Business Planning) has a significant impact for those who agreed to starting a business relative to those who strongly agreed.

We undertook a sensitivity test to assess if our results depend on our choice of the base group for the analysis by using “Disagree” as the base group. Our conclusion with regards to the University effect stands, as it shows a significant effect on “Strongly Agree” relative to “Disagree.” However, the significant impact of business planning skills disappeared in the final model. Our analysis has been focused on identifying significant factors (based on the survey conducted rather than undertaking different effects of categories or classes within our explanatory variables.

The qualitative data provide more contexts demonstrating that even with the frugal resources, students make significant gains with respect to developing basic skills and awareness. However, some barriers remain because the students struggle to apply the skills when they graduate in an environment that introduces more complexities. This suggests that the curricula and student experience should integrate more application and opportunities for real world application.

## 5 Discussion

The research demonstrates that the policy to embed entrepreneurial skills within the curricula of Higher Education Institutions (HEIs) in Nigeria is widely appreciated and considered essential by the stakeholders who engaged in the study. However, the findings suggest the enactment of the NUC policy on entrepreneurial education is currently haphazard and inefficient. Several complex and fundamental challenges are evident that make strategic implementation of the policy tedious. Even though conversations are taking place between relevant stakeholders, there needs to be a coordinated and facilitated forum which will enable co-creation at all levels and a more structured enactment strategy at institutions [72]. Therefore, one implication of the results is an urgent need to evaluate and review the effectiveness of the policy on entrepreneurship in Nigerian Universities. This should lead to a revised policy, developed by co-creation with relevant stakeholders. This updated policy should at the minimum encourage a more hands-on practical [54] element and ensure entrepreneurship and innovation are integrated and assessed across existing modules rather than the current standalone model of delivery. Integrating the triple helix model of innovation [32] which involves a university-industry-government collaboration will be highly beneficial to the achieving the positive intended outcomes.

The results, both quantitative and quantitative, indicate that the location of a university has an impact on the entrepreneurial aspirations of students. This can be attributed to the fact that proximity to high intensity commercial activities and blossoming entrepreneurial activities in large cities or hubs provides a different experience for students than small towns, similar to hub and spoke models seen with business start-ups [73]. This does not mean rural locations cannot participate in the policy with the same rigour as their counterparts. The rural or isolated HEIs will need to scaffold their provision and seek relevant and effective ways to supplement the affordances the commercial hubs provide. This can be done virtually, through locally supported hubs and collaborative ventures. Using frugal instruments and well-designed processes the more isolated HEIs can invest in a range of tools which enhance the student experience of entrepreneurship education and ensure students have authentic contact with real world experiences and can be immersed in rich entrepreneurial learning experiences. The cost of this should be reflected in support provided to identified institutions. Entrepreneurial activity hubs nests entrepreneurial education within broader sub-ecosystem of entrepreneurship [74] providing layers of opportunity for successful engagement.

It was observed that learning resources are scarce and poorly designed. To enable the policy implementation, it is important to invest in learning objects that support transformative learning experiences [75]. Resources that support authentic assessments are particularly important as the findings indicate assessments support motivation to learn [76]. Locally designed tools that account for the contemporary realities and the development of equally relevant decision making, problem solving, and resilience are specifically important [13]. Role models and stakeholders can collaboratively support the design, development and roll out of the resources across the country. The student voice in this area is essential, student reps and volunteers can help pilot and test resources. This model should be used to ensure the continuous redesign and updating of resources. Furthermore, this will be enhanced by strengthening the relationship between the town and gown (university and industry). This partnership could be encouraged by a national policy which incentivises engagement of industrial partners with universities. Similarly, HEIs should proactively seek opportunities for partnership with the industry, private organization, and other stakeholders where students could have practical exposure to innovation and entrepreneurial skills.

Learning environments need to be carefully developed to align with the policy goals and intended outcomes. Institutions need to support a holistic and scaffolded learning environment to foster entrepreneurship education [77]. On the one hand, as the study has linked the HEIs sites to motivation and readiness; it is important to understand how institutions of diverse sizes and in various locations, particularly urban and rural locations will require differing resources and links with proposed stakeholders.

It was observed that asides from the policy, there are several other opportunities students are taking advantage of to develop entrepreneurial and innovation skills, which are not necessarily provided by the university. Student capacity in this area could be developed through a range of integrated activities such as entrepreneurial master classes, development of innovation hubs, skills workshops and innovator led seminars on a wide range of areas which could shape and inspire individual interest and positively influence entrepreneurial aspirations. HEIs need to be responsive, enhancing their ability to educate and develop graduates ready for the 21st century of work. To effectively implement the NUC policy, it is imperative for innovative pedagogies to replace practices that do not support upskilling. This was also observed by Ref. [78]. The two critical dimensions identified as supporting current initiatives are well-equipped structured centres and collaborative partnerships [79]. These substantially enhance students' opportunities to acquire real world skills and inform their behaviours in relation to entrepreneurship.

Central to the need for nested entrepreneurial ecosystems and locally contextualised pedagogy, is a need to equip educators with the tools, training, and institutional support to enact the policy. Facilitator and lecturer training is central in this regard. Research around entrepreneurial education is pivotal in driving a richer entrepreneurship education experience. Training of educators ensures a holistic loop between entrepreneurs/entrepreneurial activity, stakeholders and universities are sufficiently nested in a broader entrepreneurship ecosystem. Trained educators can design relevant and up to date material and pedagogy, promote links with the communities and activities which provide real life opportunities for learning and assessment as well as provide a rich practice for research and future policy.

There are three critical pedagogic implications based on the findings of this study –

• The development of holistic learning environment,
• The use of locally contextualised learning resources,
• Training of relevant workforce.

It is important to note that these points and implications have broader application beyond entrepreneurship education. It is evident that a larger conversation around pedagogic design and innovation is desperately needed [75]. The urgent need to successfully enact the entrepreneurship policy can be an impetus for supporting pedagogic development in Nigerian HEIs.

## 6 Conclusion

The 2004 National Policy on Education (NPE) sets forth an ambitious agenda, aiming to seamlessly integrate entrepreneurship education into the curriculum across all levels. The policy underscores the imperative to cultivate entrepreneurial skills and attitudes that align with the specific needs of both the community and the national economy. Recognizing the need for relevance to local communities emphasizes the crucial role of pedagogic innovation in fostering context-specific skills and attributes. Pedagogy, as the linchpin of curriculum development and delivery, should be at the core of the NPE, ensuring that skills and competencies are imparted in accordance with national economic development goals.

This study affirms the NPE's significant contribution to promoting entrepreneurship education in Nigeria. However, while the policy has successfully raised awareness and cultivated basic entrepreneurial skills and attitudes, it falls short of advancing beyond these foundational objectives to drive economic development and translate innovation into entrepreneurial progress within the societal framework of the educational ecosystem. Evidently, the persistent challenges in the Nigerian educational landscape, including the quality and motivation of educators, insufficient infrastructure, and inadequate resources for supporting entrepreneurship education programs, hinder the realization of the policy's potential.

Moreover, this study underscores the potential of pedagogic innovation employing a frugal approach and collaboration with established stakeholders to enhance entrepreneurship education significantly. It emphasizes the necessity of creating a more entrepreneurial society by addressing deficits in the educational system. The study confirms stakeholders' willingness to collaborate with Higher Education Institutions (HEIs) but stresses the need for robust frameworks, adequate resourcing, and local support structures to catalyse hubs that promote the pathways for entrepreneurial learners to translate practice into entrepreneurial communities.

While this study offers substantial insights into the effectiveness of entrepreneurship education policy in a selected sample of Nigerian HEIs, it is not without limitations. Firstly, there were a few methodological challenges involved in collating data from a geographically widespread sample. However, this was overcome by using an online form which collated data from students relatively easily and with limited influence from the power dynamics which could skew individual opinions. Students and lecturers were keen to engage in the project and quite enthusiastic about using their voice. The researchers ensured that there was complete anonymity and confidentiality to support data collection.

Secondly, the scope of the project had to be carefully delineated as there were several dimensions and layers of data points around entrepreneurship education pedagogy in universities, innovation around engagement with stakeholders and collaboration for supporting entrepreneurial activities nationally. The project team focused on capturing a range of stakeholder engagement with entrepreneurial activities which were collated via survey and interviews. The analysis employed thematic analysis integrated with behavioral mapping. Though these instruments captured the self-reported thoughts, ideas, reflection, and behaviors of stakeholders. They are unable to support observed actions or activities. However, as many of the activities and events we are evaluating take place over many years – such as developing and implementing pedagogic change, building centres, and organising hubs and exchange programmes; a longitudinal project will be required to capture change and educational effectiveness.

Finally, the study uses data (qualitative and quantitative) from four Federal Universities in Nigeria, which is a limited number, as resources would permit. A more extensive analysis requiring a larger dataset to investigate the interaction of university with specific skills, that may affect entrepreneurial decision should be investigated. This can also facilitate the use of more robust econometrics approaches such as Two-Staged-Multinominal Analysis to control, for endogeneity, Structural Equation Modelling, amongst others. As with most qualitative studies, participant selection may not be representative. Also, private universities were not included in the study. The practices between private and public universities may significantly differ and as such, the findings in the study should be interpreted in this context. For further studies, other higher institutions of learning may be involved especially polytechnics and schools of vocational studies to have a wider and more robust result. Similarly, the perspectives of other stakeholders in the education and entrepreneurship sectors should be captured.

## CRediT authorship contribution statement

**Muyiwa Oyinlola:** Writing – review & editing, Writing – original draft, Supervision, Resources, Project administration, Methodology, Investigation, Funding acquisition, Conceptualization. **Oluwaseun Kolade:** Writing – review & editing, Writing – original draft, Validation, Supervision, Methodology, Investigation, Formal analysis. **Silifat Abimbola Okoya:** Writing – review & editing, Writing – original draft, Methodology, Formal analysis, Data curation, Conceptualization. **Olubunmi Ajala:** Writing – original draft, Visualization, Validation, Software, Methodology, Investigation, Formal analysis, Data curation. **Arinola Adefila:** Writing – review & editing, Writing – original draft, Supervision, Methodology, Investigation, Formal analysis, Conceptualization. **Adedapo Adediji:** Writing – original draft, Project administration. **Kunle Babaremu:** Writing – original draft, Data curation. **Bosun Tijani:** Writing – original draft, Supervision, Resources, Funding acquisition, Data curation, Conceptualization. **Jude Adejuwon:** Writing – original draft, Resources, Project administration, Conceptualization. **Faith Wambui:** Writing – original draft, Project administration, Data curation. **Esther Titilayo Akinlabi:** Writing – review & editing, Supervision, Funding acquisition.

## Declaration of competing interest

The authors declare the following financial interests/personal relationships which may be considered as potential competing interests: Muyiwa Oyinlola reports financial support was provided by 10.13039/501100000308British Council London.
