# Supplementary material for: Entrepreneurship and innovation in Nigerian universities: Trends, challenges and opportunities
Source: Heliyon. 2024 Apr 23;10(9):e29940. doi: 10.1016/j.heliyon.2024.e29940 (PMC11070808; doi:10.1016/j.heliyon.2024.e29940)
Supplement: Multimedia component 1 [file mmc1.docx]

**Supplementary Material for**

**Entrepreneurship and Innovation in Nigerian Universities: Trends, Challenges and Opportunities**

Muyiwa Oyinlola^a*^, Oluwaseun Kolade^b^, Silifat Abimbola Okoya^a^, Olubunmi Ajala^c^, Arinola Adefila^d^, Adedapo Adediji^e^, Kunle Babaremu^e^, Bosun Tijani^f^, Jude Adejuwon^f^, Faith Wambui^g^, Esther Titilayo Akinlabi^h^

^a^ Institute of Energy and Sustainable Development, De Montfort University, Leicester, UK, LE 1 9BH

^b^ Sheffield Business School, Sheffield Hallam University, Sheffield UK

^d^ School of Economics, Finance and Accounting, Coventry University, CV1 5DL

^d^ Staffordshire Centre for Learning and Pedagogic Practice ST4 2DE.

^e^ Directorate of Pan African University Life and Earth Sciences Institute (PAULESI), Ibadan, Oyo State, Nigeria

^f^ Co Creation Hub, 294 Herbert Macaulay Way, Yaba, Lagos Nigeria

^g^ iHub, Galana/Lenana Road, Nairobi, Kenya

^h^ Department of Mechanical and Construction Engineering, University of Northumbria, Newcastle upon Tyne, UK

*Corresponding Author: [Muyiwa.oyinlola@dmu.ac.uk](mailto:Muyiwa.oyinlola@dmu.ac.uk)

## Survey questions

1. Gender [Male/Female]
2. Age ([18-24]; [25-34]; [35-44]; [45-54]; [55-64]; [65-74]; [75 and over])
3. Location (State and(or) LGA)
4. What qualification are you studying for? [Undergraduate, Masters, PhD, Primary, Secondary, Other]
5. What is your employment Status (Part-time work, Full-time work, Self Employed/run a business, Others(specify:.....)]
6. Field of study [Engineering, Agriculture Arts, Humanities, Medicine, Sciences, Computer Science, Other (specify) ….]
7. Name of University [Unilag, PAULESI, ABU,  OAU, UNN, other (Specify)]
8. Are you currently running a business? (Y/N)
9. Have you ever been part of a business that contributes to the circular plastic economy? [yes, no]
10. How do you rate your ability to prepare a business plan? (None, low, moderate, high, excellent)
11. How do you rate your ability to generate financial value (e.g. income/profit) from a circular plastic product? (None, low, moderate, high, excellent)
12. How do you rate your ability to generate financial value (e.g. income/profit) from a circular plastic service? (None, low, moderate, high, excellent)
13. How do you rate your ability to generate business solutions that address societal problems? (None, low, moderate, high, excellent)
14. How do you rate your user engagement skills? (None, low, moderate, high, excellent)4
15. How do you rate your ability to market your products/services? (None, low, moderate, high, excellent)
16. How do you rate your ability to build a team for your business venture (None, low, moderate, high, excellent)
17. How do you rate your ability to lead a team in your business venture?  (None, low, moderate, high, excellent)
18. How do you rate your book-keeping (managing cash inflow and outflow) skills?  (None, low, moderate, high, excellent)
19. Have you started a business? [Yes/ No]
20. Have you been a part of a group that started a business? [Yes/ No]
21. After my studies, I would like to start my own business [  strongly disagree, disagree, neither disagree nor agree, agree, strongly agree,]
22. I prefer to work for a business than start my own [ strongly disagree, disagree, neither disagree nor agree, agree, strongly agree]
23. What options will you consider to fund your business [Bootstrapping (savings, friends or family), Grants, Loans (bank, microfinance, convertible e.t.c), Crowdfunding, Venture Capitalist, Business incubator/accelerator, other please specify]  multiple answer option
24. I want to start a business that contributes to the circular plastic economy.   [strongly disagree, disagree, neither disagree nor agree, agree, strongly agree,]
25. I want to work with  a business that contributes to the circular plastic economy [  strongly disagree, disagree, neither disagree nor agree, agree, strongly agree,]

## Focus group discussion questions

1. Introductions
2. What is the university doing to help student acquiring entrepreneurial skills? Follow up to appraise responses
3. What is the university doing to help student be innovators? Follow up to appraise responses
4. What other things have helped you in your entrepreneurial and innovation journey? Follow up to appraise responses
5. What can the university do to enhance student skills for innovation and entrepreneurship?
